# Chest-to-Back Skin-to-Skin Contact to Regulate Body Temperature for Low Birth Weight and/or Premature Babies: A Crossover Randomized Controlled Clinical Trial

**DOI:** 10.1155/2021/8873169

**Published:** 2021-04-26

**Authors:** Sisay Gere, Yemane Berhane, Alemayehu Worku

**Affiliations:** ^1^Department of Nursing, College of Health Sciences, Arsi University, Asella, Ethiopia; ^2^Department of Epidemiology and Biostatistics, Addis Continental Institute of Public Health, Addis Ababa, Ethiopia; ^3^Department of Reproductive Health and Population, Addis Continental Institute of Public Health, Addis Ababa, Ethiopia; ^4^Department of Biostatistics, Faculty of Health, Addis Ababa University, Addis Ababa, Ethiopia

## Abstract

Skin-to-skin contact (SSC) is one of the critical components of kangaroo mother care (KMC), which is an intervention to enhance the survival of low birth weight (LBW) and/or premature infants in low-income settings. Chest-to-chest (CC) contact has been practiced widely; however, mothers face practical challenges to continuously provide CC-SSC. Hence, we assessed the efficacy of chest-to-back (CB) SSC as an alternative to CC-SSC in regulating body temperature for LBW and/or premature babies in Ethiopia. We applied a noninferiority clinical trial among LBW and/or premature infants admitted to a referral hospital neonatal intensive care unit (NICU) in Ethiopia. The study randomized the infants into two crossover arms; arm 1 applied first CB-SSC followed by CC-SSC, and arm 2 applied first CC-SSC followed by CB-SSC. The outcome measure was a change in skin temperature. We used a linear mixed-effect model for analysis. The result showed no statistically significant difference in the mean temperature between the comparison arms. In conclusion, we found that the CB-SSC was not inferior to the CC-SSC in regulating body temperature of the babies. Thus, CB-SSC can be further investigated as an alternative to CC-SSC in the kangaroo care model in low-income settings.

## 1. Background

Kangaroo mother care (KMC) has been advocated for neonatal care in low-income settings since 1979 [1, 2]. KMC helps to regulate physiological stability, improves growth and development, promotes infant-to-parent bonding, and reduces morbidities and mortalities of infants born prematurely and/or with low birth weight [3–5]. KMC is a feasible and cost-effective neonatal care package especially for low-income settings [6–8]. Although there is significant heterogeneity in the definition of KMC, the skin-to-skin contact (SSC) is a core and universally accepted component of the KMC [9]. SSC is critical for low birth weight (LBW) and/or premature babies that are at higher risk of hypothermia [10–12]. Skin-to-skin contact is commonly provided through chest-to-chest contact of the mother and her baby [6, 13].

The continuous SSC in KMC, which is sustained over a long period of time, is essential especially for LBW and or premature infants [13, 14]. Ideally, SSC must begin at birth and continue without interruption until the time that it is no longer needed, which could run for several months [3, 6, 15]. Such prolonged CC-SSC pose practical challenges once the mother resumes daily chores after birth [16, 17]. These practical barriers, besides the weaknesses in the health system, have made the KMC to be underutilized in low-income countries, where it is needed most [18–20]. Similar challenges were observed in Ethiopia [21, 22]. The practical challenges to utilizing CC-SSC include cultural beliefs such as “baby should be carried on the back,” not on the chest [17, 23], and the inability of mothers to perform daily chores in forward inclination position [17, 24, 25]. Carrying the baby on the chest hinders physical activities for mothers whose livelihood relies on farming, daily labor, and other physically demanding tasks [17, 26, 27]. Thus, mothers are unlikely to adhere to CC-SSC for a long period of time once discharged from the hospital care. In Ethiopia, about 60% of deaths in preterm infants, who received KMC in hospitals, occur after discharge from the hospital [14].

Thus, assessing an alternative SSC option that is culturally acceptable and practically feasible is essential for a successful and widespread implementation of KMC in low-income settings. Carrying infants on the back is a common and culturally acceptable practice by mothers in low-income settings [17, 28]. Thus, research on chest-to-back SCC is imperative in low-income settings where morbidity and mortality attributed to hypothermia are high and implementing CC-SSC remains low [19, 21, 29] due to practical challenges. This study was conducted with the objective of assessing the efficacy of CB-SSC in regulating body temperature for LBW and/or premature infants in Ethiopia. Our hypothesis was that the CB-SSC is not inferior to CC-SSC in regulating body temperature for LBW and/or premature infants.

## 2. Materials and Methods

### 2.1. Trial Design

The design was a randomized control crossover clinical trial.

### 2.2. Study Setting

The trial was conducted in Asella Teaching and Referral Hospital, Neonatal Intensive Care Unit, in Ethiopia. The hospital is run by Arsi University, and its catchment population is estimated to be more than 3.5 million [30]. The number of deliveries per year in the hospital was estimated to be 8400. Respectively, the neonatal ICU and the KMC room had 26 and 4 beds. Annually, about 1500 babies were admitted to the neonatal ICU ward. But the annual number of admission to the KMC room was about 30. One of the discharging criteria from the KMC room was baby weight. The baby has to weigh at least 1800 grams to be discharged, given that he/she has no other serious health problems. Thus, very low birth weight babies will stay in the KMC room until they weigh 1800 grams. Generally, babies admitted to the KMC room will stay for two to three weeks before discharge, whereas babies admitted to the neonatal ICU will stay for a week. Therefore, there were 7-8 admissions/bed/year in the KMC room and 58 admissions/bed/year in the neonatal ICU. The nurse to patient ratio was 1 : 8 in addition to the two pediatricians who were in charge of the ICU and KMC services [30]. The study period was prolonged until the sufficient number of babies admitted to the KMC room was obtained for this study.

### 2.3. Participants

**Figure 1:**
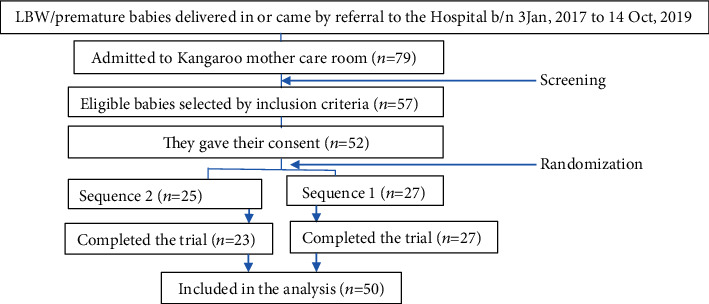
Participant flow.

The participants of this study were LBW (<2500 grams) and/or premature (<37 completed weeks of gestation) infants (Figure 1). To determine their weight eligibility for the trial, they were weighed naked with a digital weighing scale of a 10-gram interval, whereas their gestational age eligibility was estimated by using the Ballard score or the last menstrual period if the mother knew her last menstrual period. Participants were excluded from the study if they had malformations or birth disabilities, or if they had been dependent on oxygen or IV fluid, or if they had any other serious disorders [31, 32]. Furthermore, babies whose gestational age was not at least 32 weeks and/or babies whose weight was less than 1000 grams at enrollment were excluded (see Figure 1).

### 2.4. Ethical Considerations

This trial was approved by the Arsi University Ethical Review Committee (reference number A/CHS/RC/15/16). The trial was also registered in ClinicalTrials.gov (NCT04346498). Informed written consent was obtained from participants. We conducted the trial as per the approved protocol, and according to the good clinical practice guidelines and the national ethical guidelines, and in accordance with the Helsinki declaration.

### 2.5. Randomization

Participants were allocated into two sequential enrollment schemes by randomly assigning the “odd” and “even” days of a month into sequence 1 or sequence 2, respectively. That is, based on “born on odd day” and “born on even day” of a month. Participant enrollment continued in that sequence until we achieved the sample size.

### 2.6. Intervention

**Figure 2:**
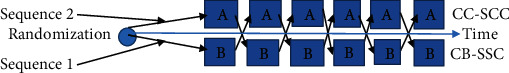
Schematic representation of a multi period—ABABAB/BABABA crossover trial.

As summarized in Figure 2, the trial compared CB-SSC and CC-SSC periods in a crossover design. For CB-SSC, the naked chest of a newborn was positioned upright on the naked back of the mother between the two scapulae in a direct SSC. For CC-SSC, the naked chest of the newborn was positioned upright in a direct SSC on the naked chest of the mother between her breasts. Babies were kept in either position alternatively (crossover) every two hours while their temperature was registered for 3 consecutive days for each pair. There was a 1-hour washout or rest period before the baby was crossover to the next SSC (see Figure 2).

To minimize the between-subject variability, all study newborns were provided the same kind of diaper, warm hat, and socks. Babies were also wrapped on the mother with the same kind of cloth. The trial was conducted in the daytime. Furthermore, the chronological age of the newborn (the age at which the newborn received the intervention), the weight of the newborn on those intervention days, skin temperature of the mother, and the room temperature during those intervention days were recorded. Chronological age was measured in days. Weight was measured using a digital scale of 10-gram intervals. We weighed the newborn naked before feeding. Room temperature was measured in degree Celsius using a device named the Wall Clock with Temperature and Humidity Indicator. The device was donated to the neonatal ICU and KMC room of our study site by UNICEF. It measures temperature range from negative 30 to positive 50 degrees Celsius. The brand name is Brannan. Brannan 28/600/0 has also the ISO certification and quality management approval. It was from Brannan Thermometer Cleator Moor Cumbria, England [33]. Skin temperature of the mothers was measured in degree Celsius. To measure the skin temperature of the chest of the mother, the probe was placed about 6 to 7 cm away from the sternal notch to the body of the sternum (between her breasts). For the back, temperature was measured on the spine between the superior angle of the right scapula and the superior angle of the left scapula. Furthermore, pulse oximetry recording was done continuously in the infants in both groups.

**Figure 3:**
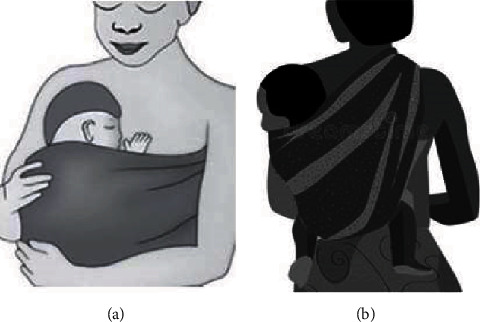
Pictorial representation of kinds of skin-to-skin contacts. (a) Represents the CC-SSC, and (b) represents the CB-SSC.

The intervention was performed by trained female study nurses. In either method of the SSC, the position of the mothers was sitting. They sit on their bed, and the bed was characteristically normal (it was not reclining). Figures 3(a)–3(b) represent the CC-SSC and the CB-SSC positions, respectively. (But see the supplemental materials (available here) (the supplemental procedure or protocol, the videos (video for CC-SSC and video for CB-SSC), and the photos) for more and detail understanding about the intervention.)

### 2.7. Outcome Measure

We measured the outcome by measuring the skin temperature changes of the newborns at 10-minute intervals of 2 hours. Over 3 days of stay in the trial, the newborns in the study had 78 measurements. Out of these 78 measurements, 6 were taken as the baseline (3 for the experiment and 3 for the control period) and 72 were taken after the baseline measurements (36 for the experiment and 36 for the control period). We used a monitor called the Multi-Parameter Patient Monitor to measure the skin temperature of our infants and the mothers as well. Its brand/trade name is CONTEC (from the CONTEC CO., LTD subsidiaries in China (Contec Solution China Corporation). Address: No.112 Qinhuang West Street, Economic & Technical Development Zone, Qinhuangdao, Hebei Province, China). According to the company, its products have passed CE, FDA, and COS/VIOS certificate (FDA&CE ICU CCU Vital Signs Patient Monitor, 6 Parameters, CMS, 8000) [34]. The monitor measures skin temperature in the range of 0°C to 50°C with ±0.1°C precision. The monitor has a probe. To measure the skin temperature of the newborns, we placed the end of the probe (i.e., the sensor) just between the two scapulae of a baby. This is to mean, the sensor was placed amid the inner end upper curve of the left scapula and the inner end upper curve of the right scapula. The site was the same for both the experimental and control arms. The SI unit used was degree Celsius. (For the detail, see supplemental materials.)

### 2.8. Sample Size

The sample size estimation was done based on the following assumptions: comparison arm not worse than -0.5°C, alpha 0.05, power 90, and standard deviation of 0.86 [35]. Accordingly, our calculated sample size per group was 50 mother-baby pairs. To maximize the effect difference detection efficiency of the trial, we took repeated measurements from each study subject, 78 times per subject. Repeated measures increase the power of the study with a single outcome measure by decreasing the standard error of the treatment effect [36].

### 2.9. Statistical Analysis

Data entry and cleaning were done in EPi Info software. The data analysis was done in SPSS version 21. The data were restructured in a long format and transformed into base 10 logarithms for analysis. Thus, analysis was done to examine the changes observed in 10-minute intervals. We used a linear mixed-effect model to estimate the effect difference between the two arms. In the model, diagonal variance-covariance structures and autoregressive moving averages were fitted to analyze the random effects and the effects of the repeated measures in that order. A model that had smaller Akaike information criteria was chosen, and the noninferiority *t*-statistics (TNI) = (Mean difference (CB‐SSC‐CC‐SSC) + (the priori defined worse))/standard error [36] was computed and compared against alpha 0.05. Additionally, a *t*-test was used where necessary.

## 3. Results

The flowchart is presented in Figure 1. Out of 57 eligible baby-mother pairs, 52 (91·23%) offered informed written consent and enrolled in the trial though 2 withdrew from the study after consenting. Thus, 50 completed the trial as per our protocol. The baseline characteristics of the newborns are depicted in Table 1. On average, they entered into the trial in their second week of birth (Table 1).

The mean skin temperature of the mother was 33.25°C for the chest and 33.37°C for the back. There was no statistically significant difference in the skin temperature of the chest and the back of the mother (Table 2). Table 2 also shows the ambient room temperature recorded during the control and experimental phases of the trial; the range was 6.70°C and 10.20°C, respectively.

The mean skin temperature of the newborns when they began the SSC and when they end up with the SSC is depicted in Table 3. At either time point, no difference was noted between the arms, indicating that there was no significant effect difference between the arms (Table 3).

Table 4 shows the summary of the effects (the skin temperature changes) observed in our study babies. The 5% trimmed mean (95% CI) was 0.114 (0.106, 0.122) for the CB arm and 0.127 (0.116, 0.138) for the CC arm, indicating no statistically significant difference (Table 4).

**Figure 4:**
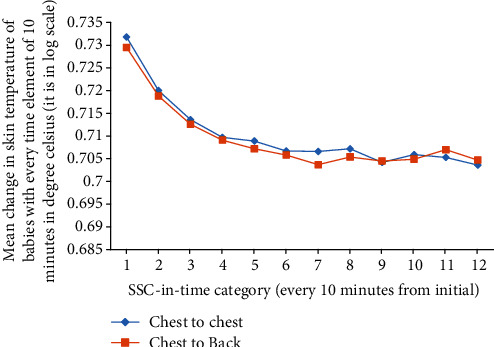
The distributional curve for the effect of CB-SSC and CC-SSC by time.

As shown in Figure 4, in either group, the effect observed on skin temperature of our patients had not been constant over time. It rather decreases in size in an exponentially decaying linear order from the initial. That is, improvement was effected on our patients by either type of the SSC.

Adjusted for all covariates, the pairwise comparison test of our linear mixed-effect model illustrated that the CB-SSC and the CC-SSC have no significant effect differences between them (Table 5).

From Table 5, the calculated noninferiority *t*-statistics (TNI) was 499. So, when 499 was compared to the priori *α* (0.05) level (approximate value 1.678) with *t* distribution at 1 − *α* = 0.95 and degree of freedom = *n*1 + *n*2 − 2 (27 + 23–2) 48, 499 is greater than 1.678 (*P* < 0.0001). Thus, the null hypothesis defined at a priori worse level “Warming LBW and/or premature infants by the CB-SSC is ≥0.5°C lower than with warming by the CC-SSC” was rejected.

**Figure 5:**
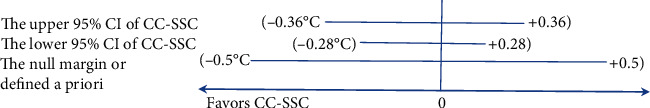
Comparison of the 95% CI of the comparator's SD (current result) versus the priori defined worse.

Assuming that larger outcomes are better, if we perform discounting on the TNI result and preserve the standard deviation of the active comparator in Table 4 above to determine the noninferiority margin, the result would be 0.318 (95% CI: 0.278 to 0.363).  Figure 5 compares this margin, which preserved the substantial part of the efficacy of the active control (72% preserved) with the priori defined worse level (-0.5°C). Again, the worse, -0.5°C, was not included by this margin.

### 3.1. Trial-Associated Adverse Effects and Complications

In this study, we did not come across trial-associated adverse effects, major complications, and/or deaths.

## 4. Discussion

This study demonstrates that the chest-to-back SSC was not inferior to the chest-to-chest SSC in regulating the body temperature of the babies in this study.

The observed finding corroborates many other studies that showed that the chest and the back of an adult human being have uniform thermal comfort [37] although the upper back of the body of an adult human being has higher skin temperature than that of the front side [38–40]. Due to a lot of blood flow to the heart, the area of the chest that is closer to the heart has a higher skin temperature [39]. In general, the skin temperature on the chest of an adult human being ranges from 30.9 to 36.1°C, and that of the back ranges from 32.4 to 36.3°C [37]. In this study, the mean skin temperature of the chest (measured between the two breasts of the mothers) was found to be 33.254 degrees centigrade, whereas the mean skin temperature of the upper back (measured between the two scapulae of the mothers) was 33.377 degrees centigrade. No statistically significant difference was noted between the chest and the back skin temperature of the mothers (*P* > 0.05), which verifies that the skin temperature differences between the front and the back are minimal and are not considered physiologically meaningful [40]. Thus, as long as appropriate dressing and prolonged contact are maintained, either the chest or the back of the mother can provide sufficient thermal body regulation for the baby [40].

Heat exchange occurs between the skin surfaces of the mother/caregiver and the baby by a process of heat exchange called direct conduction [41]. As such, the amount of heat conduction may not have meaningful differences whether the child is in contact with the mother on her chest or her back, which supports our result that showed noninferiority of the CB skin-to-skin contact as compared to CC skin-to-skin. Our finding indicates both CB and CC approaches may be used to regulate the body temperature of low birth weight and/or premature infants. Moreover, both approaches equally fulfill the desired attributes for skin-to-skin contact in low-income settings, which include humanism, naturalism (i.e., both of them are natural ways of warming), being cheap, availability, and accessibility [5, 8, 42]. Hence, the CB-SSC does not compromise the benefits which are attributed to the CC-SSC.

In fact, the CB-SSC has additional benefits as it is more culturally acceptable to the local population. The CB care provides more freedom to the mother to participate in income-earning activities and social events. The CB is just normal and thereby less stigmatizing; commonly, there is a stigma associated with having a baby with special needs [23]. In the Ethiopian culture, mothers are accustomed to wrapping infants on their back, especially when travelling and performing their daily activities [17]. This facilitates the successful adoption, diffusion, and utilization of the SSC [43]. Thus, adding the CB-SSC as an alternative to care may enhance the uptake of KMC and improves the survival of babies, which is also in line with the Alma Ata declaration that emphasizes respect to the culture of the community [44].

The key concept behind the SSC is to maintain thermal balance both in hospital and postdischarge until the baby is capable of self-controlling his/her own body temperature [6, 13, 45]. This is because LBW and/or premature babies lack sufficient fat to allow them to control their body temperature, which often exposes them to hypothermia [32, 46] regardless of the weather and climatic conditions [47]. Thus, it is important to keep the SSC in all settings and under all circumstances [13] in order to safeguard vulnerable babies from the risk of hypothermia [48]. In low-income settings, health care providers have been reporting inability to achieve thermal control only by using the CC-SSC due to challenges related to adhering to this approach by the mothers [13, 49]. Hence, our study that showed the noninferiority of the CB-SSC gives hope to provide an alternative approach for maintaining a prolonged SSC in low-income settings where mothers have multiple roles in society apart from just providing care for the newborn. Furthermore, enhancing adherence to SSC using either CC-SSC or CB-SSC can help to save more lives than what was possible by the CC-SSC alone. This would further lessen the costs that the family and the health care delivery system and/or the country can expend, because neither of them requires heavy investment apart from proper health education and counseling [50].

Finally, in countries where adherence to the CC-SSC is low [19, 29, 51], this study provides compelling evidence that the CB-SSC is not inferior to CC-SSC; in addition, CB-SCC is more culturally acceptable and is less likely to interfere with routine responsibilities of the mothers. As this is the first study that investigates the effect of CB-SSC in Ethiopia, however, additional studies should be warranted before scaling up. Moreover, we did not assess the long-term survival of babies. Hence, future works should also include follow-up designs at both facility and home levels. Our trial babies were on average in the second week of life, and they may be better stable than freshly born babies. Thus, the method should be tested on freshly born babies in future studies. The crossover trials have their own drawbacks [52, 53] as well; thus, future research needs to consider this limitation when designing studies. Last but not least, this is a small-scale trial, which was conducted in a single hospital; thus, we recommend a multicenter study with a larger sample size.

## 5. Conclusion

In conclusion, the CB-SSC was not found to be inferior to the CC-SSC in regulating temperature of low birth weight and premature babies in this trial. We recommend further multicenter and community-based studies to attest to the noninferiority of the CB-SSC approach and its feasibility to overcome some practical challenges associated with the implementation of the CC-SSC approach and enhance adherence in low-income settings.

## Figures and Tables

**Table 1:** General characteristics of babies.

Characteristics	Mean ± SD or median (Q1, Q3) or frequency (%)
Birth weight (gram)	1466.4 ± 201.6
Gestational age at birth (weeks)	33.7 ± 1.3
Small for gestational age	
Yes	11 (22)
No	39 (78)
Weight on first trial day (gram)	1466.4 ± 184.1
Age on first trial day (days)	15.5 (9, 21)
Sex of babies	
Girls	26 (52)
Boys	24 (48)

**Table 2:** Skin temperature of the chest and the back of the mothers and room temperature.

Variables	Statistics	Trial arms			
		Chest (in °C)	Back (in °C)	*t*-statistics	*P* value
Skin temperature of mothers	Mean	33.254	33.377	1.202	0.231
	Median	33.50	33.60		
	Mode	34.00	34.00		
	Std. Dev.	1.629	1.556		
Room temperature	Mean	26.20	26.28		
	Minimum	22.0	21.5		
	Maximum	28.7	31.7		
	Std. Dev.	1.61	2.02		

**Table 3:** Mean skin temperature of newborns when measured in both the groups at the beginning of SSC and at the end of SSC.

Trial arm	Skin temperature in °C at the beginning of the SSC			Skin temperature in °C at the end of the SSC		
	Mean (SD)	*t*-test	*P* value	Mean (SD)	*t*-test	*P* value
CC-SSC	34.82 (0.82)	0.35	0.72	36.88 (0.51)	0.59	0.55
CB-SSC	34.53 (0.96)			36.92 (0.59)		

**Table 4:** Descriptive statistics for the skin temperature change per arm.

Descriptive statistics	CB (°C)	CC (°C)
Minimum value	-5.00	-2.10
Maximum value	3.40	4.70
Median (interquartile range)	0.1000 (0.20)	0.1000 (0.20)
Standard deviation	0.297	0.318
5% trimmed mean (95% CI: lower, upper)	0.114 (0.106, 0.122)	0.127 (0.116, 0.138)

**Table 5:** The pairwise comparison output of our final linear mixed-effect model.

Intervention category		Mean difference in °C	SE	df	Sig	95% CI in °C	
(I)SSC	(J)SSC						
CC	CB	0.001	0.001	16156.964	0.143	.000	0.002
CB	CC	-0.001	0.001	16156.964	0.143	-.002	0.000

## Data Availability

Upon a reasonable request, we can offer access to the datasets used and/or analyzed. This can be available from the corresponding author.

## Supplementary Materials

Supplementary Materials This paper has supplemental materials. Thus, interested readers can also refer to supplementary materials (procedure, video, and photo) for detailed understanding. The procedure or protocol consists of detailed written procedures that were used during the trial. The photos show the CB-SSC kangarooed LBW and/or premature infant and the CC-SSC kangarooed LBW and/or premature infant. The video clips display when the study nurses were carrying out the intervention (the CB-SSC and the CC-SSC).
