# Supplementary material for: Chest-to-Back Skin-to-Skin Contact to Regulate Body Temperature for Low Birth Weight and/or Premature Babies: A Crossover Randomized Controlled Clinical Trial
Source: Int J Pediatr. 2021 Apr 26;2021:8873169. doi: 10.1155/2021/8873169 (PMC8096581; doi:10.1155/2021/8873169)
Supplement: Supplementary Materials — This paper has supplemental materials. Thus, interested readers can also refer to supplementary materials (procedure, video, and photo) for detailed understanding. The procedure or protocol consists of detailed written procedures that were used during the trial. The photos show the CB-SSC kangarooed LBW and/or premature infant and the CC-SSC kangarooed LBW and/or premature infant. The video clips display when the study nurses were carrying out the intervention (the CB-SSC and the CC-SSC). [file 8873169.f1.zip › Suplemental Procedure or Protocol for Manuscript8873169-HIJP.docx]

**አርሲ ዩኒቨርሲቲ
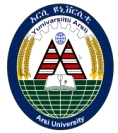
 ARSI UNIVERSITY**

**www.Arsiuniversity.com**

**Phone +251-223313429**

**Fax +251-223313430**

**P.O. BOX 193 Asella, Ethiopia**

**LIKE OUR STAR ATHLETES EXCELLING PROFESSIONALS!**

**Protocol/Procedure To Execute The Chest-to-Back And The Chest-to-Chest SSC**

To mentor and perform the trail efficiently, the protocol is categorized in to 2 phases. Phase one is called the preparatory phase and phase two is called the intervention phase.

**Phase I: The Preparation Phase**

Step 1. LBW and/or premature infants admitted to KMC room

A. ***SCREENING*** [Critical, don’t miss any item of the screening template]

Step 2. Ask the mother or caregiver consent to screen the baby for eligibility

Step 3. If the mother or the care giver provides you an approval, then, provide information about “why you screen?” (Use the screening information sheet)

Step 4. Then, conduct screening (use the screening template)

Step 5. If the baby is not eligible, thanks the mother and family, and provide the usual care

Step 6. If the baby is eligible, then, re-request the second consent for detail explanation of the whole nature of the study. If you are not received this consent, thanks the mother and family, and provide the usual care

Step 7. If you received the second consent, provide detail explanation about the whole nature of the study (Use the trial information sheet)

Step 8. Then, receive a signed consent if the mother agrees to participate on the trial with her newborn baby (Use the Consent form). If not, thanks the mother and her family and stop to proceed. However, you have to continue to provide the usual services.

Step 9. If you received the signed consent at step 8, then, continue to pretest activities

***B. PRE-TEST ACTIVITIES***

Step 10. Entry of the consent signed participant into the pretest stage

Step 11. Together with the mother, set a schedule. The schedule should be for 3 consecutive days, performed for 2 hours a day per each type of the SSC (total 4 hours). Including rest time, the schedule should be for 5 hours per day for 3 consecutive days (Grand total, it is a 15 hours schedule).

Step 12. Ahead of the scheduled days, you have to make sure that all equipment and supplies required for the study are available.

Step13. One day ahead from the schedule, perform the following activities:

A. Collect basic data of the baby and the mother (Use the basic data collection format).

B. Educate the mother about SSC (Use the teaching guideline). If possible, include the family

C. Check that all the devices and the monitors to be used for the study are functional

D. Check the availability of the clothes to be used for the study (the nappy, the socks, the cape, the T-binder, the pajama/ gown, the scarf and so on). They must be those clothes that are prepared for this study.

E. Check that formats or templates, stationaries, and other supplies are ready and functional.

F. Ensure the random assignment (check it by using the cross-checkup method you trained).

G. As a team, are you all (the study nurses, the mother, and the supervisor) okay to enter to the intervention? 0. No, ----- 1. Yes, ------- If No, get back and do what is left. If yes, tick on ‘yes’ and proceed to the intervention phase by tomorrow, as scheduled.

NB: The mother (with her baby) has the right to withdraw from the trial at any point in time without any jeopardy upon her, her baby, and her family too.

**Phase II: The Intervention Phase/ Executing the Skin-to-Skin Contact**

In order to help to perform and monitor the activities smoothly and rightly, this phase is divided in to 6 parts (This is excluding part 7). Start to preform part 1 and part 2 as of some 20 -30 minutes ahead of to apply the SSC.

**Part 1:**

Step 1. Bring the equipment and supplies to the KMC room

Step 2. Arrange all equipment to the bedside. Possibly, in the order you will use them.

Step 3. Perform the necessary installation activities, such as electric power divider installation and so on.

Step 4. Check the random group of the baby. Is it A or B? If “A” get ‘template “A” series with you. If “B” get ‘template “B” series with you

Step 5. Is it the first day, -------- second day------ or third day-------? (Tick on the Template)

Step 6. Is it period 1----2----3-----4----5-----6------? (Tick on the Template)

**Part 2:**

Step 7. Weight the baby naked and Record it (use template-3).

Step 8. Dress the baby with Nappy, Cape, Socks, and T- binder.

Step 9. Feed the baby and record it (Use template-4).

Step 10. Measure and record Feeding, Skin T^0^, Pulse, Respiration, and O_2_ of mother. (Use template-4). NB: Site to put/place/ the Skin T^0^ probe:

For chest: 6 to 7 cm from the sternal notch towards the body of the sternum.

For Back: Between the superior angles of right and left scapula [Centrally = on the spine].

Step 11. Remove the monitor from mother or care giver

Step 12. Secure the skin T ^0^ probe between the superior angles of right and left scapula of the baby [Just at the center= on the spine]. This means, just between the inner end upper curve of left scapula and the inner end upper curve of right scapula

Step 13. Secure the Pulse Oxymetery probe/sensor on the foot of the baby.

**Part 3: PLACING the BABY in to the SSC**

Step 14. Use protocol A to put the baby into CC-SSC and protocol B to put the baby in to CB- SSC

| *PROTOCOL-A: Protocol to place the baby into Chest-to-Chest SSC* |
| --- |
| 1. Request the mother to sit comfortably on her bed  2. Undress the baby gently, except for cap, nappy, socks and T-binder  3. Place the baby prone on mother’s chest in an upright position between her breasts, in skin-to-skin contact (mainly the face, chest, abdomen, arms and legs to remain in SSC) with baby’s arms flex, hips also flexed and extended like a “FROG”; turn baby’s head to one side in a slightly extended position to keep airways clear and look up the MOM too  4. Secure the baby thoroughly with the T-binder  5. Cover the baby with mother’s gown or pajama, which was prepared for this study purpose.  6. Wrap baby-mother together with an added scarf or “Fota”, which was prepared for this study purpose.  7. Keep the baby in this position for 2:00 hours unless otherwise indicated  START Recording!!!! Wrapping with Scarf or “Fota” is the point to plug the probe and sensor to their respective machine and to start recording parameters [Use template-5] |

| *PROTOCOL-B: Protocol to place the baby into Chest-to-Back SSC* |
| --- |
| 1. Request the mother to sit comfortably on her bed  2. Undress the baby gently, except for cap, nappy, socks and T-binder  3. Place the baby prone on mother’s Back in an upright position between her scapula’s, in a skin-to-skin contact (mainly the face, chest, abdomen, arms and legs to remain in SSC) with baby’s arms flex, hips also flexed and extended like a “FROG”; turn baby’s head to one side in a slightly extended position to keep airways clear and look after the baby too.  4. Secure the baby thoroughly with the T-binder  5. Cover the baby with mother’s gown or pajama, which was prepared for this study purpose.  6. Wrap baby-mother together with an added scarf or “Fota”, which was prepared for this study purpose.  7. Keep the baby in this position for 2:00 hours unless otherwise indicated.  START Recording!!!! Wrapping with Scarf or “Fota” is the point to plug the probe and sensor to their respective machine and to start recording parameters [Use template-5] |

**Part 4: Intra SSC**

Intra SSC is the time from the start of the SSC (0 hour) to end of the SSC. It has a 2 hours length. This is a very critical period. Important activities during this intra SSC period are:

Step 15. Reassess the correctness of the SSC you have done in step 14 above (Use template-6).

Step 16. Monitor the parameters of the baby continually.

Monitor the skin T^0^ continually and recorded it (Use template 5).

Monitor Pulse, O_2_, and respiration continually and record it (Use template 5).

Monitor the sleep pattern (Use template 7).

Step 17. Monitor the room temperature continually and record it (Use template 8)

Step 18. Also, monitor the whole process continually. In the meantime, if you found any abnormal conditions or any risks or danger signs (especially with respect to the baby), then, take an immediate action! Don’t wait a minute! (Use template 9 and 8 accordingly).

Step 19. After you completed the first 2 hours, then, the washout or rest period will follow.

**Part 5: Washout Period**

This period has 1 hour. There is no SSC at this time. Important activities during this time are:

Step 20. Conducts the assessment required for this period and record the data (use template 5)

Step 21: Continue the usual service for the baby and the mother

Step 22. Do all the preparations (including step 8 to 13) for the SSC you will perform following the end of the washout period.

**Part 6: The CROSS Over**

Step 23. After completion of the washout period, crossover the baby to the other type of the SSC. Now, depending upon random sequence (sequence A or Sequence B) of the baby, perform either step 14-A or 14-B. Then, proceed to step 15 to 18. NB: This is the second 2 hours.

**Part 7: End of the SSC of the Day**

After the end of the second 2 hours (means 2 hours as of you crossover the baby), the day’s trial activity was completed. Now do the following:

Say ‘Thank you!’ for the mother and the family for all they have done. They deserve.

Conduct the post- assessment and record the data (use template 5)

Assemble or collect the material and supplies used; and check them for your next day activities

Continue the usual service for the baby and the mother

Have a brief meeting with your superior (or significant others if any) and set the way forward for your next day activities and close-up the session.
