# Supplementary figures and images for: Chest-to-Back Skin-to-Skin Contact to Regulate Body Temperature for Low Birth Weight and/or Premature Babies: A Crossover Randomized Controlled Clinical Trial
Source: Int J Pediatr. 2021 Apr 26;2021:8873169. doi: 10.1155/2021/8873169 (PMC8096581; doi:10.1155/2021/8873169)

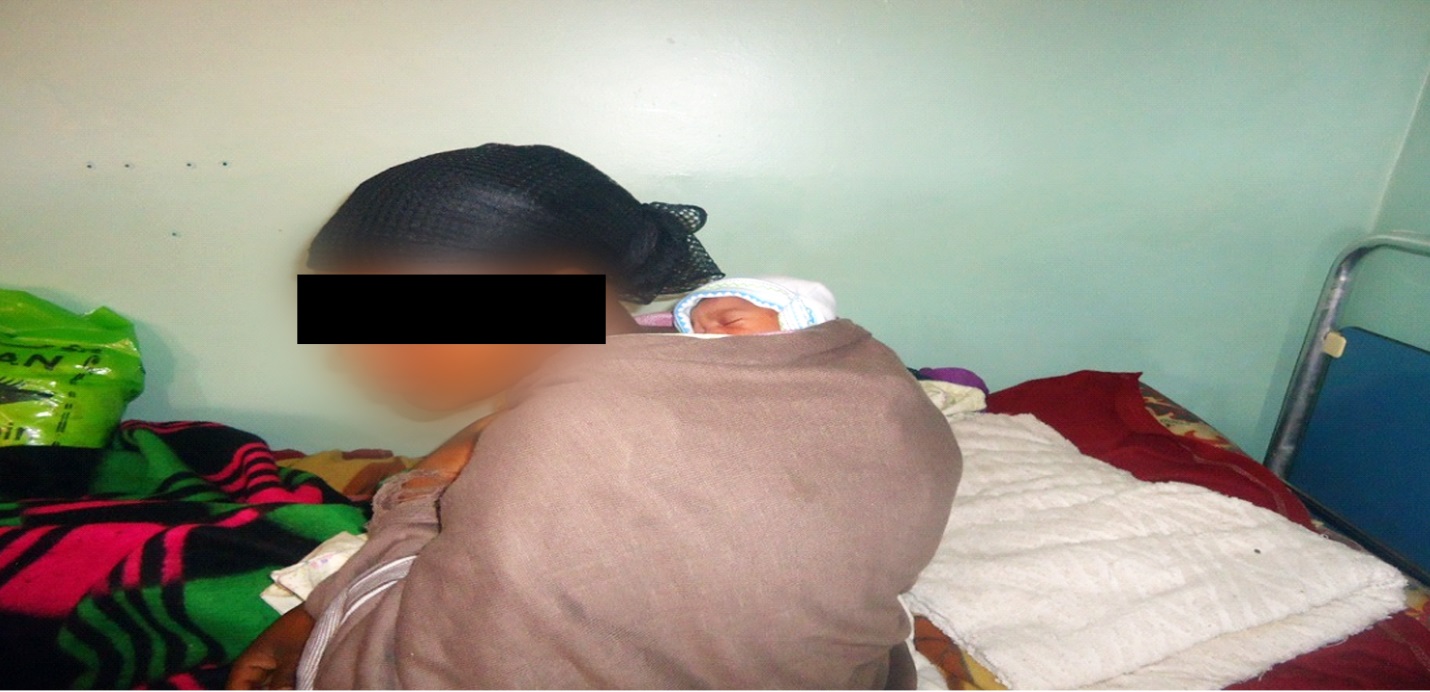


1. The CB_SSC


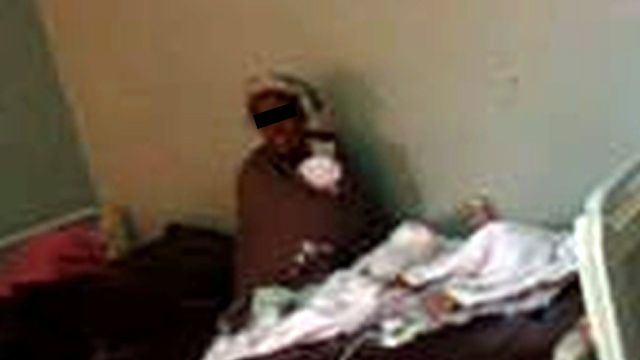


1. The CC-SSC

Supplement: Supplementary Materials — This paper has supplemental materials. Thus, interested readers can also refer to supplementary materials (procedure, video, and photo) for detailed understanding. The procedure or protocol consists of detailed written procedures that were used during the trial. The photos show the CB-SSC kangarooed LBW and/or premature infant and the CC-SSC kangarooed LBW and/or premature infant. The video clips display when the study nurses were carrying out the intervention (the CB-SSC and the CC-SSC). [file 8873169.f1.zip › Suplemental Photo.docx]
